# Behavioral and stress responses to feeding time in pregnant sows under limit-fed regime

**DOI:** 10.1093/jas/skab108

**Published:** 2021-04-08

**Authors:** Hayford Manu, Suhyup Lee, Mike C Keyes, Jim Cairns, Samuel K Baidoo

**Affiliations:** 1 Southern Research and Outreach Center, University of Minnesota, Waseca, MN, USA; 2 Department of Swine and Poultry Science, Korea National College of Agriculture and Fisheries, Jeonju 54874, South Korea; 3 Remote Insights Management Solutions (RIMS) Inc., Minneapolis, MN, USA

**Keywords:** behavior, cortisol, feeding time, isocaloric intake, pregnant sows

## Abstract

We investigated the effect of feeding time on behavior and stress responses in pregnant sows under isocaloric conditions. Twenty-four sows were balanced for parity and randomly assigned to 1 of 3 feeding times. Corn–soybean meal-based diet was fed once at: 0730 (Control, T1), 1130 (T2), and 1530 hours (T3). On average, sows received 7,062 kcal ME/d from 2.20 kg of diet formulated to contain SID Lys/ME of 1.71 g/Mcal. The study was conducted for 28 d (21 d acclimation to the feeding regime and 7 d data collection). Saliva samples were collected every 2 hr for 12 hr in stalls on day 52 of pregnancy. Behavior data were collected 24 hr for 7 d from day 53 of gestating by affixing a remote insights ear tag to each sow. Each sow had 120,960 data points categorized into: “Active,” “Feed,” or “Dormant”. Due to housing constraint, all sows were housed in individual stalls in the same barn presenting a potential limitation of the study. Data were analyzed using PROC MIXED and GLIMMIX procedures of SAS 9.4 for cortisol and behavior data, respectively. Sow was the experimental unit. The area under the curve (AUC) is quantitative evaluation of response as threshold varies over all possible values. A 12-hr cortisol total AUC for sows fed once daily at 1130 hours was reduced relative to sow group fed at 1530 hours (*P* = 0.046) but similar compared with the control sows (*P* = 0. 323). The control sows (0730 hours) had reduced total (*P* < 0.001) and feeding (*P* = 0.001) activity AUCs relative to sows on 1130 hours but did not differ compared with sows on 1530 hours feeding schedules (*P* > 0.100). Sows on 1130 hours feeding schedule had greater feed anticipatory activity, 24-hr total activity count, total (*P* < 0.001) and feeding (*P* < 0.001) activity AUC compared with sows fed daily at 1530 hours. In conclusion, feeding pregnant sows earlier in the morning (0730 hours) appears to minimize sows’ behavior but similar cortisol response. Sows on 1130 hours feeding schedule had greater activities but reduced cortisol concentration, suggesting that elevated sow activity might not necessarily indicate activation of hypothalamic–pituitary–adrenal axis.

## Introduction

Nutritional effects in many mammalian models differed by the timing of food intake under isocaloric conditions (Noeske-Hallin et al., 1985; Arble et al., 2009; Nikkhah, 2012; Wang et al., 2014). Feeding time elicited a response on sow’s performance under limit-fed conditions (Manu et al., 2019). However, the welfare implication associated with such feeding times on stress and sow behavior under limit-fed conditions remains to be elucidated since provision of feed within a narrow time window each day leads to significant changes in physiology and behavior (Johnston, 2014). Nevertheless, behavioral studies to evaluate animal welfare are characterized by scan sampling (Robert et al., 2002; Hwang et al., 2016; Basavaraju et al., 2017) and single time point cortisol measurements (Hemsworth et al., 2016; Li et al., 2017). Although acceptable, such procedures may not provide a complete evaluation leading to loss of information if biological reasoning behind the scan sampling and single time cortisol measurements are not known. Sow welfare is a concern 24 hr/d.

Feeding behavior can be measured by meal duration, feeding rate, and feeding time (Bargo et al., 2018). However, without holding total caloric intake constant, meal-timing data cannot be the useful (Kulovitz et al., 2014). To the best of our knowledge, no information is reported about total and feeding activity of sows in response to feeding time under isocaloric conditions. As part of a lager project to understand the impact of feeding time in pregnant sows, we hypothesized that feeding the same amount of energy per kilogram live BW^0.75^ at different time of day will increase the activation of the HPA axis and adversely affect pregnant sows’ behavior. The objective of this study was to investigate the effects of feeding time based on similar energy intake per kilogram live BW^0.75^ on sow’s stress response, feed anticipatory activity (FAA), and 24-hr feeding and total activities of pregnant sows.

## Materials and Methods

### Animals, housing, and management

The study was performed at the swine unit of University of Minnesota Southern Research and Outreach Center, Waseca, MN. University of Minnesota Institutional Animal Care and Use Committee (IACUC) approved all protocols used in the study IACUC No: 171011961.

**Figure 1.**
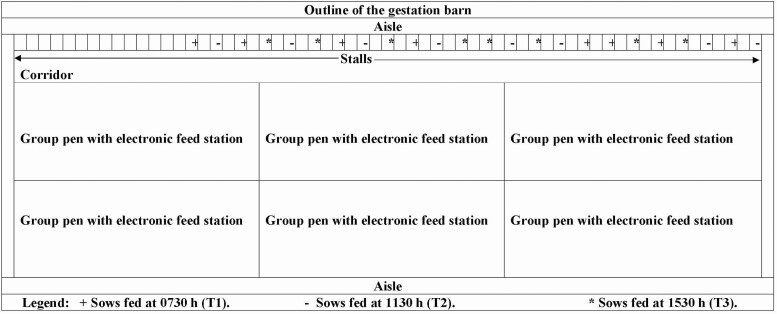
Sows were maintained in individual stalls. An empty stall separated 2 adjacent sows. Sows in stalls were fed once daily at 0730 hours (T1, +), 1130 hours (T2, −), and 1530 hours (T3, *).

Saliva cortisol concentrations were measured on day 52 of pregnancy from nulliparous and multiparous pregnant sows of Topigs Norsvin (Landrance × Large White); total *N* = 24; 8 sows per treatment; parity 3.13 ± 0.56; and BW 217.04 ± 4.35 kg. The stalls for individual housed pigs were 2.1 m × 0.59 m × 0.97 m (2.04 m^2^) with fully slatted floor under temperature-controlled environment (22 ± 1 °C). Within experimental sows, there was an empty install between adjacent sows to prevent direct body contacts (Figure 1). Light was turned on at 0730 hours and off at 1630 hours daily to provide a 9-hr light and 15-hr of dark schedule. Sow’s body weight and backfat thickness were measured using an ultrasound machine (Lean-Meater, Renco Corp., Minneapolis, MN) before feeding, on the day 30 of gestation. Measurements were taken at the last rib about 5 cm lateral from the dorsal midline on both left and right sides using oil as coupling fluid and the 2 readings averaged. Sows had ad libitum access to water through nipple drinkers fitted to each pen. From weaning through breeding to 30 d, feed offered to sows was restricted to 2 kg, which is considered optimal for gilt and sows during that period of production.

### Experimental design, dietary treatments, and feed line calibration

A complete randomized design was used in this study. Pregnant sows were balanced for parity and randomly allocated to 1 of 3 feeding times with 8 replicates per treatment. All sows received a common corn–soybean meal-based diet from days 30 to 60 of gestation. Nutrients met or exceeded NRC 2012) nutrient requirements for pregnant sows. Experimental treatments were imposed from 30 d of gestation with 21 d adaptation to the feeding schedules. Body weights on day 30 were used to adjust the amount of feed fed between days 30 and 60 of gestation. To standardize ME intake per kilogram live BW^**0.75**^, the daily quantity of feed fed was scaled to the BW^**0.75**^ live weight (Le Naou et al., 2014) and fed at 1.25 times (Prunier and Quesnel, 2000) the maintenance requirements for sows (100 × BW^**0.75**^ kcal ME/d; NRC, 2012). On average, sows received 7,062 kcal ME/d from days 30 to 60. To provide a daily energy intake, sows received on average 2.20 kg from days 30 to 60 of gestation. Sows were fed individually by raising the feeder ball valve of an Accu-Drop feed dispenser (AP Systems, Assumption, IL) to drop the required amounts of feed into the feeding troughs. The Accu-Drop feed dispensers were calibrated at the day 30 at various set points and related the volume of Feed Dispenser, *Y* (cm^3^) to kilogram weight of feed (*x*) delivered as: *Y* = 5.4864*x* + 1.9087; *R*^2^ = 0.9892. The required daily feed allowance was provided once daily at: 0730 (Control, T1), 1130 (T2), and 1530 hours (T3).

### Saliva sample collection as biomarker of stress response

Each sow was sampled 7 times from 0630 to 1830 hours. Saliva samples were collected 1 hr before and after each feeding time and 3 hr after the last feeding occasion (i.e., 0630, 0830, 1030, 1230, 1430, 1630, and 1830 hours) using neutral synthetic swab Salivette (Sarstedt, Aktiengesellshaft and Co, Numbrecht, Germany) attached to cotton string. The string was hung in the stall to allow sows to chew on the salivette until it became completely soaked with saliva (Greenwood et al., 2016). Saliva samples were collected on ice and centrifuged 2 hr later at 2,500 × *g* for 10 min at 4 °C. Approximately 0.5 mL saliva was obtained from each swab and frozen at –20 °C until required for analysis of cortisol concentration.

### Saliva cortisol analysis

Saliva samples were analyzed for cortisol in duplicate using commercially available ELISA kit (Neogen Corp., Product number 402710, Lexington, KY) according to the manufacturer’s instructions. The ELISA was validated for recovery and parallelism with swine saliva as previously described (Li et al., 2017). The minimum detectable concentration of cortisol was 0.04 ng/mL and the intra- and interassay coefficient of variation were 8.4% and 12.2%, respectively. To minimize interassay variations, samples from all treatments and same time points were analyzed within the same assay.

### Collection of sow behavior data

A subset of multiparous and nulliparous pregnant sows of [Topigs Norsvin (Landrance × Large White); total *N* = 18; 6 sows per treatment; initial average BW 223.19 ± 3.85 kg and average parity 3.72 ± 0.57] from the stress physiology study were sampled for sow behavior studied from d 53 of gestation for 7 d without human interference, although daily animal checks were carried out by farm attendance during feeding times. One technology to assess behavior of sows is the Remote Insight Sow Management Solution (RISMS) system (Manu et al., 2020). The RISMS is designed to identify sows’ events of interest 24 hr for any number of days. The RISMS consist of a wireless ear tag that monitors sow movements. The ear tag data are intermittently sent to a gateway in the barn that forwards it to Google’s Cloud Platform where the behavior data are process using machine learning models. Briefly, the data were collected by affixing a remote insights ear tag to each pregnant sow. The ear tag sent 3 axis accelerometer data in x, y, and z plane collected at 2 Hz to a cloud database. The raw accelerometer data were then passed through a machine learning model which classified the activity of the sow per epoch (epoch length = 5 s) into 1 of 3 broad categories: “Active,” “Feed,” or “Dormant” (Table 1). This resulted in 120,960 data points per sow over the 7 d study period after 21 d adaptation to the feeding regime. The data were aggregated and reported every 15 min for 24-hr to minimize the noise in the data set. The results presented are mean 15 min epoch “Feed” and/or “Active” classifications per sow over 7 d. Pigs fed at different times of the day could not be housed in different rooms within the barn. To minimize this expected impact on our results, we allowed 21 d adaptation before any data collection. In addition, experimental sows in adjacent stalls were separated from each other with and empty stall, Figure 1.

**Table 1.** Ethogram of gestating sows’ behavioral activity

Type of behaviour	Description of behavior
1. Total FAA	The average of all feeding related activity 1-hr before feeding (de Godoy et al., 2015); average per 15 min
2. Total feed activity	The number of 5 s periods that the model detected feeding behavior (eating and/or sham chewing); average value per 15 min for 24 hr.
3. Total activity	The number of 5 s periods that the model detected sow movement; average value per 15 min 24 hr

To record feeding activity, the ear tag accelerometer captures the movement of the head. The sow’s head has a distinct gyration when chewing. The model currently cannot distinguish between sham-chewing and the chewing of feed. “Active” behavior or activity is movement excluding “Feed” behavior and small motions such as dream tremors and very brief movements of the head (e.g., to shake a fly off).

### Determination of FAA in sows

FAA in all sows was recorded every 15 min as feeding activity 1-hr prior to the scheduled feeding times. Total daily FAA was the sum of all feeding activity 1-hr prior to each feeding time (de Godoy et al., 2015).

### Validation and precision of the machine learning model

To validate the machine-learning model, video of sow behavior was labeled for the distinct behavior categories (feeding, active, and dormant) and corresponding ear tag accelerometer data was used to train and test the machine-learning model to identify when those behaviors occurred. The precision of the machine-learning model is measured as a percent confidence. For each sow, we used 72, 576 data points (60%) representing 4.2 d to develop model and 48, 384 data points (40%) from 2.8 d to test the model. The precision of the model was ~94% confidence.

### Chemical analysis and feed composition

Feed samples at the feed mill and during feeding were pooled for analysis. The DM, crude protein, crude ash, NDF, and ADF were analyzed by the methods previously described (Manu et al., 2019). Basically, the diet was corn and soybean based (4,431 kcal/g GE, 15.7% crude protein, 13.3% NDF, and 4.80% ADF).

### Calculation of cortisol and behavioral activities area under the curve (AUC)

Cortisol AUC (ng*hr/mL) and behavior count AUC (count*hr) were calculated for 12 hr and 24 hr, respectively, using trapezoidal summation rule:∑{[(*Ct* + *Ct*+1) × 0.5] × Δ*I*}; where *C*_*t*_ is either the concentration of a saliva cortisol in nanograms per milliliter or behavior count of an animal at time t, and for the next data *C*_*t+1*_, with a time interval of Δ*I* in hours between data points, and ∑ is the sum of the responses from *C*_*t*_ to *n* − 1 total number of data time points (Veissier et al., 2001).

### Statistical analysis

Statistical analyses were performed using SAS version 9.4 (SAS Inst., Inc., Cary, NC). Data normality was checked using the PROC UNIVARIATE procedure. Sow behavior count and AUC data were not normally distributed. Log transformation using the equation ((*X*’3 = log 10(*X* + 0.5) + 0.5) was adopted to achieve variance homogeneity (Hwang et al., 2016). The log transformed count behavior, FFA, and AUC data were analyzed by fitting a logistic model using the GLIMMIX procedure. Back transformed results were reported. Cortisol data taken over seven time points were analyzed as repeated measures ANOVA using the PROC MIXED procedure of SAS (SAS Inst. Inc., Cary, NC). The model included fixed effects of treatment, time, and treatment × time interaction with sow as random effect. Autoregressive process of first order was used to model repeated observation within sow as covariate structure (Littell et al., 1998). Adjustment to the denominator of degree of freedom was determined by the Kenward-Roger’s method (Kenward and Roger, 1997). Differences in basal cortisol concentration at 0630 and at 1830 hours were compared using a 1-sided paired test with PROC T-Test in SAS. Cortisol AUC, basal, 1, 3, and 5 hr postprandial cortisol concentration data were analyzed by the PROC MIXED procedure of SAS. All pairwise differences of least square means were evaluated with the PDIFF option of SAS and adjusted for multiple comparison by the Tukey–Kramer method. Sow was the experimental unit in all analysis. Statistical significance and tendencies were declared at *P* < 0.05 and *P* ≤ 0.10 for all statistical tests, respectively.

## Results

### A 12-hr cortisol response to feeding time under isocaloric intake in pregnant sows

The least square means of 12-hr salivary cortisol concentrations in pregnant sows with respect to feeding time are presented in Table 2. Interaction of cortisol concentration by time was not significant (*P* = 0.202). Treatment 1, 2, and 3 had peak cortisol concentrations of 0.66, 0.69, and 0.68 ng/mL, respectively, at baseline (0630 hours) but there was no difference among treatments (*P* ≥ 0.10). Similarly, feeding time did not alter cortisol concentrations at 1030, 1230, 1430, 1630, and 1830 hours (*P* > 0.10). Mean cortisol level was affected by time with concentration at 0630 hours being greater than 1830 hours (0.677 vs. 0.449 ng/mL; *P* = 0.026). The 12-hr cortisol total AUC for sows fed once daily at 1130 hours was reduced relative to sow group fed at 1530 hours (*P* = 0.046) but similar compared with the control (*P* = 0. 323). Feeding sows once daily at 1530 hours did not alter the 12-hr cortisol AUC relative to the control sows fed daily at 0730 hours (*P* = 0. 479).

**Table 2.** Cortisol concentration, cortisol AUC, and probability values of pregnant sows subjected to different feeding times under limit-fed regime

	Time of day, hours							
Item	0630	0830	1030	1230	1430	1630	1830	Total AUC, ng*h/mL^4^
Trt. 1, 0730 hours^1^	0.663	0.698	0.457	0.319	0.372	0.345	0.484	326.02^ab^
Trt. 2, 1130 hours^2^	0.686	0.390	0.423	0.302	0.324	0.281	0.471	272.15^b^
Trt. 3, 1530 hours^3^	0.682	0.388	0.459	0.357	0.564	0.450	0.394	349.10^a^
SEM	0.09	0.08	0.09	0.07	0.08	0.08	0.09	57.21

^1^Sows received their daily gestation ration once at 0730 a.m.

^2^Sows received their daily gestation ration once at 1130 a.m.

^3^Sows received their daily gestation ration once at 1530 p.m.

^4^Total AUC was calculated using the trapezoidal summation method.

^a,b^Least square means in each column followed by different superscripts significantly differ (*P* < 0.05; Tukey–Kramer adjusted).

### Basal, pre-, and postprandial cortisol concentrations and AUC with reference to feeding time

Least square means of basal, pre- and post-prandial cortisol concentrations and AUC with reference to feeding time are presented in Table 3. The 3-hr post-prandial cortisol concentrations and 1-hr pre-prandial cortisol levels were not affected by feeding time (*P* ˃ 0.10). Feeding time affected 1-hr post-prandial cortisol concentration with sows fed at 1130 hours having lower values (*P* = 0.014) relative to the control sows but did not differ compared with sows fed at 1530 hours (*P* = 0.458). The control sows (0730 hours) and sows receiving their daily ration at 1530 hours did not differ in cortisol levels 1-hr after feeding (*P* = 0.131). The 5-hr post-prandial cortisol concentration did not change for sow groups fed at 0730 and 1130 hours (*P* = 0.744). The 3-hr (*P* = 0.030) and 5-hr (*P* = 0.020) cortisol AUC was lower for sow receiving their daily ration once daily at 1130 hours compared with the control sows. The 3-hr cortisol AUC was not different between the control sows and sows fed daily at 1530 hours (*P* = 0.192). Likewise, sows on 1130 and 1530 hours feeding schedule had similar 3-hr cortisol AUC (*P* = 0.527).

**Table 3.** Basal, pre-, and postprandial cortisol concentrations, and AUC with reference to feeding time^1^

	Treatment				
Variable	T1^2^	T2^3^	T3^4^	SEM	*P*-value
Time 0, baseline	0.65	0.71	0.67	0.14	0.873
1 hr before feeding	0.65	0.42	0.56	0.09	0.135
1 hr after feeding	0.70^a^	0.30^b^	0.45^ab^	0.08	0.017
3 hr after feeding	0.45	0.32	0.38	0.07	0.429
5 hr after feeding	0.31	0.29	* ^5^	0.08	0.744
AUC^6^, ng*h/mL	108.41^a^	68.65^b^	83.62^ab^	13.24	0.036
AUC^7^, ng*h/mL	156.33^a^	98.36^b^	* ^5^	20.16	0.020

^1^Total area under the curve (AUC) was calculated using the trapezoidal summation method.

^2^Sows received their daily gestation ration once daily at 0730 hours.

^3^Sows received their daily gestation ration once daily at 1130 hours.

^4^Sows received their daily gestation ration once daily at 1530 hours.

^5^Sow group fed at 1530 hours could not be sampled 5 hr after feeding.

^6^AUC from time 0 to 3 hr after feeding.

^7^AUC from time 0 to 5 hr after feeding.

^ab^Least square means within a row with uncommon superscript significantly differ (*P* < 0.05; Tukey–Kramer adjusted).

### Behavioral activity of pregnant sows in response to time of feeding under isocaloric intake

**Figure 2.**
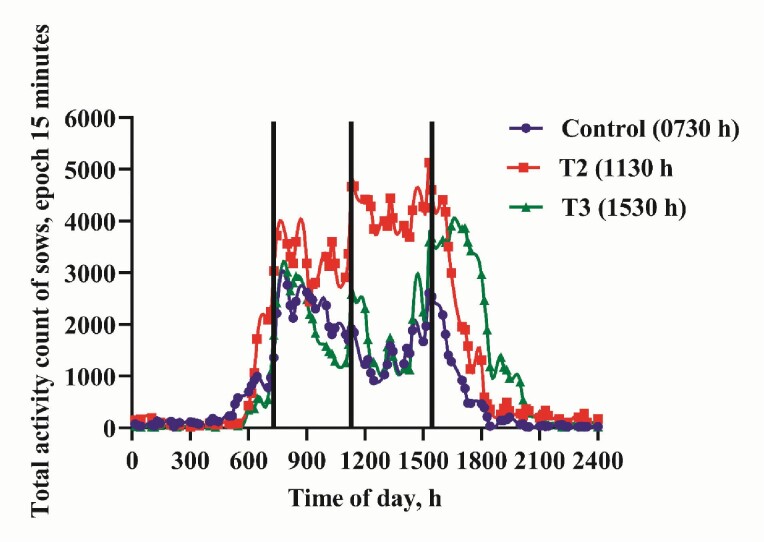
Temporal pattern of pregnant sow’s total activity measured for 24-hr period over 7 d under limit-fed regime. The graph represents sows fed once daily at 0730 hours (T1, violet curve), 1130 hours (T2, red curve), and 1530 hours (T3, green curve). The vertical black lines indicate feeding times for each treatment group.

**Figure 3.**
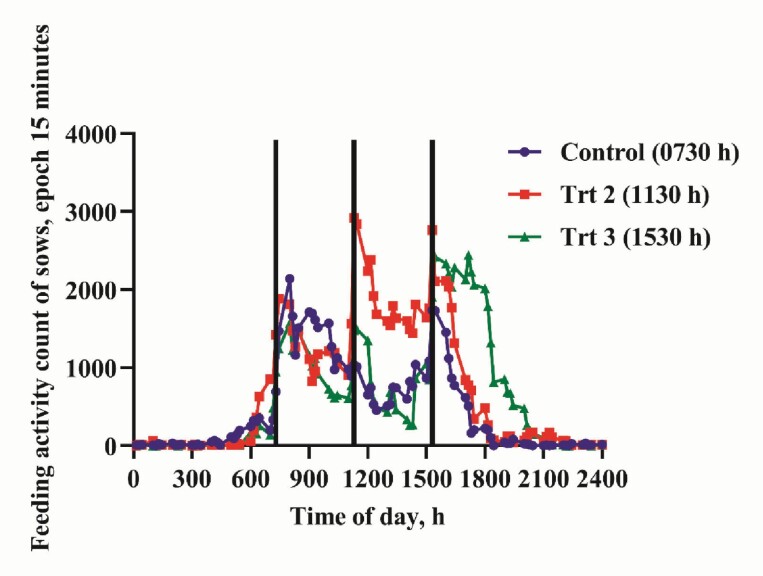
Temporal pattern of 24-hr feeding activity profile of pregnant sows fed at different times of the day for 7 d under limit-fed regime. The graph represents sows fed once daily at 0730 hours (T1, violet curve), 1130 hours (T2, red curve), and 1530 hours (T3, green curve). The vertical black lines indicate feeding times for each treatment group.

Following the 21-d adaptation to the feeding regimes, the behavioral activities in response to feeding time are presented in Table 4. Sows fed daily at 0730 hours had lower 24-hr total activity count compared with sows fed daily at 1130 hours (*P* < 0.001) but similar to sows on 1530 hours feeding schedule (*P* > 0.100; Figure 2). Feeding sows at 1130 hours daily resulted in greater 24-hr total activity (*P* < 0.001) but similar total feeding activity (*P* = 0.051) relative to sows fed daily at 1530 hours. A 24-hr total feeding activity was lower with sow fed at 0730 hours than sows receiving their daily ration at 1130 hours (*P* < 0.001) but similar to 1530 hours sow group (*P* = 0.265; Figure 3). Sows on 0730 hours daily feeding schedule had lowest FAA compared with treatment group fed at 1130 and 1530 hours (*P* < 0.001). Also, the 1130 hours treatment group had greater FAA relative to sows fed at 1530 hours (*P* < 0.001). The total activity and feeding activity AUCs mirrored the FAA of pregnant sows. The control sows (0730 hours) had reduced 24-hr total (*P* < 0.001) and feeding (*P* = 0.001) activity AUC relative to sows on 1130 and 1530 hours feeding schedule, respectively. Sows on 1130 hours feeding schedule had greater 24-hr total (*P* < 0.001) and feeding (*P* < 0.001) activity AUC compared with sows fed daily at 1530 hours. Total activity of the sows started to increase from 0530 hours and nadir around 1900 hours (Figure 1). Total activity of sows on 1530 hours schedule nadir at 2000 hours. The control sows (0730 hours) had 2 peaks of total activity at 0730 and 1530 hours. Sows fed at 1130 hours had highest total activity peak at each feeding time followed by sows fed daily at 1530 hours. Sows on 1530 hours feeding schedule had intermediate peak at each feeding time (Figure 2). Feeding activity of the sows started to increase from 0530 hours and nadir around 1900 hours. Feeding activity of sows on 1530 hours schedule nadir at 2000 hours. All treatment groups had peak feeding activity at their respective feeding times, but sow group fed at 1530 hours had extended peak feeding activity, which lasted for about 3 hr. Sows fed at 1130 and 1530 hours had 3 peaks while the control sows had 2 peaks of feeding activity.

**Table 4.** Pregnant sows activity counts and AUC in response to feeding time under limit-fed condition (geometric mean [95% confidence interval])^1^

	Treatment			
Variable	T1^2^	T2^3^	T3^4^	*P*-value
Total activity counts/15 min	98.9^a^ (90.2–108.5)	197.1^b^ (182.7–212.5)	124.4^a^ (111.8–138.4)	<0.001
Total feeding activity counts/15 min	25.2^a^ (22.0–28.8)	50.3^b^ (44.0–57.5)	33.7^ab^ (28.9–39.3)	<0.001
Total FAA^5^ counts/hr	95.1^a^ (90.2–100.3)	373.5^b^ (354.3–393.7)	207.4^c^ (195.1–220.4)	<0.001
Total activity AUC^6^, counts*hr	340,565.0^a^ (335,737.6–345,461.8)	682,495.8^b^ (682,495.8–692,309.0)	497,851.7^c^ (489,666.1–506,174.2)	<0.001
Total feeding activity AUC^6^, counts*hr	182,095.8^a^ (179,060.6–185182.5)	262,361.4^b^ (247,913.4–277651.5)	223,614.5^c^ (206,300.4-242381.8)	<0.001

^1^Least square means were calculated from transformed data and then back-transformed for presentation of data.

^2^Sows received their daily gestation ration once at 0730 a.m.

^3^Sows received their daily gestation ration once at 1130 a.m.

^4^Sows received their daily gestation ration once at 1530 p.m.

^5^Total FAA was the sum of every 15 min feeding activity 1-hr preprandial.

^6^Total area under the curve (AUC) was calculated using the trapezoidal summation method.

^a–c^Least square means within a row with uncommon superscript letters significantly differ (*P* < 0.05; Tukey–Kramer adjusted).

## Discussion

Provision of feed within a narrow time window each day leads to significant changes in physiology and behavior (Johnston, 2014). This study tested the hypotheses that provision of isocaloric diet per kilogram live BW^0.75^ at different time of the day could alter stress response, FAA, and 24-hr feeding and total activity of pregnant sows. Due to housing constraint, animals on different treatments could not be housed in different rooms within the barn and the sound of feeding could have stimulated feeding behavior in the other sows. This presents a potential limitation of the study. However, to reduce this expected impact on our results, experimental units were evenly distributed between stalls, making sure experimental sows are not next to each other. Additionally, 3 wk adaptation period to the feeding regimes was allowed before data collection.

The basal concentrations of cortisol observed at 0630 hours for the various treatments groups in the morning were higher than values obtained at 0830 hours. Cortisol secretion follows circadian rhythm and appears to be biphasic with elevated levels in the morning which gradually decline across the remainder of the day (Sunaina et al., 2016). This secretary pattern was evident in our study with the greater cortisol concentration observed at 0630 hours relative to 0830 hours which is in agreement with earlier reports (de Jong et al., 2000; Hemmann et al., 2012; Amdi et al., 2013). This indicates that our sampling protocol, study design, and feeding time did not alter the circadian regulation of cortisol. We can also infer that feed intake in pregnant sows is not necessary to invoke peak cortisol concentrations since all treatment groups had peak levels preprandial. Therefore, neural, circadian, and behavioral factors associated with feed presentation may play a role to determine peak cortisol concentrations. Further, the current study also gives credence to the consensus that single point measurements of glucocorticoids have little biological value in interpreting the level of individual stress in mammals (Hanninen et al., 2006; Medica et al., 2011), even though our cortisol data presented may be related to sow activity in a particular feeding regime.

Calorie restriction may alter some stress-related pathways by enhancing basal glucocorticoids concentrations (Levay et al., 2010; Kenny et al., 2014). Levay et al. (2010) demonstrated a dose–response relationship between calorie restriction and corticosterone in rats such that the greater the calorie restriction, the higher the basal circulating corticosterone concentration. Because sows in this study had similar caloric intake per kilogram live metabolic weight (BW ^**0.75**^) irrespective of their feeding regime, the basal cortisol concentrations did not differ amongst treatments. However, sows fed daily at 1130 hours had reduced cortisol AUC relative to sows fed daily at 1530 hours but similar to the control sows. This observation is in agreements with the results of other investigators who studied cortisol rhythmicity in human volunteers in relation to meal time (Legler et al., 1982). We theorized that the 1130 hours meal may at least have a synchronizing role on plasma cortisol diurnal variations. Our data provide evidence that feeding time influence the daily plasma cortisol pattern, but no clue was found as to why feeding the same amount of energy per kilogram live BW^0.75^ at different time of day will affects the hypothalamic–pituitary–adrenal axis differently. Conversely, feeding sows once daily at 1130 hours resulted in an increased sow total and feeding activity (Figures 2 and 3) but lower cortisol AUC compared with other treatments groups. Although actions of glucocorticoids may impact animal welfare, the relationship between glucocorticoids and quality of life is dynamic and complex. Therefore, using peripheral measures alone to provide clues about welfare is limited in scope. For instance, Ralph and Tilbrook (2016) demonstrated that gluconeogenesis in laying hens was upregulated only when corticosterone in the liver was increased and glucose was depleted. Therefore, they concluded that a change in peripheral corticosterone was not an indicative of the effect of the stressor on the welfare of the hens. Additionally, Jensen et al., (1996) reported that the effects of stress on physiological and behavioral data can be contradictory. They explained that plasma cortisol concentrations of pigs submitted to unpredictable and unavoidable electric shocks for 30 d did not differ from control animals that experienced no shocks; although the behavior of the treatment group suggests that they were still stressed by the electric shocks.

Total activity and feeding activity of the sows in this study did not return to baseline from 0600 to 1800 hours. This observation was expected. The remote insight’s sow management model detected increased sows’ total and feeding activity between 0530 and 1900 hours for sows fed at 0730 and 1130 hours. Feeding sows at 1530 hours extended this period by 1 hr. The pig is investigatory in nature and they explore their surroundings by rooting, sniffing, or chewing on objects during their active time (Studnitz et al., 2007). Our observation is in agreement with the findings of Rijnen et al. (2003) who reported that pigs are most active from 0700 to 2200 hours. Sows in the current study had restricted amount of conventional diet for optimum production and sow longevity. However, considering the feeding activity pattern of the sows is possible that the feeling of fullness was not met during the day. For instance, sows on the 0730 hours feeding schedule had increased feed activity at 0730 hours and a gradual decline over time. During the second feeding time 1130 hours (4 hr after feeding), the 0730 hours fed sow had reduced feeding activity. But after 8 hr of feeding, sows on 0730 hours feeding schedule had increased feeding activity probably they might have entered into the postabsorptive state.

Sow groups fed at 0730 and 1130 hours had peak feed activity at their respective feeding time which declined drastically 1-hr postprandial. This was not the case with sow group fed daily at 1530 hours. The drop in their feeding activity from the peak was minimal and remained elevated for about 3 hr after feeding. Brouns et al. (1994) reported that sows that are restrictively fed searched for feed for at least 1 hr after they had consumed their feed. The reason why sows fed daily at 1530 hours had about 3 hr of extended feeding activity is difficult to explain and merits further investigation. It could be speculated that feeding sows once daily at 1530 hours modulated their feeding and activity behavior around the time of feeding and the performance response of feeding sows once daily at 1530 hours was previously reported (Manu et al., 2019). Treatments groups fed at 0730, 1130, and 1530 hours had peak feeding activities at their respective feeding times relative to other times of the day. This suggests that the sows adapted well to their feeding schedule. Also, the daily total activity pattern was similar among treatments and peaked around feeding times, with sows fed at 1130 hours having activity counts above the 0730 and 1530 hours fed groups for most of the 24-hr period. Feeding activity pattern followed the same trend except that at 0730 hours, the control sows had greatest feeding activity peak. Overall, the control sows had lower total and feeding activity pattern with reference to treatment group fed at 1530 hours.

Behavioral activities preceding food provision is termed food anticipatory activity (Johnston, 2014). Pregnant sows on 0730 hours feeding schedule had lowest 1-hr total FAA compared with other feeding times. Although all treatment groups had increased FAA prior to their schedule feeding times (data not shown), sows fed at 0730 hours had only one additional FAA prior to 1530 hours feeding time, whereas sows fed at 1130 and 1530 hours had 2 additional increased in FAA at (0730, 1530 hours) and (0730, 1130 hours), respectively. This additional increase in FAA was more pronounced in sows on 1130 hours feeding regime which cumulatively resulted in greatest 1-hr total FAA. This is the first study to report the effect of feeding time on 1-hr total FAA, 24 -h feeding and total activity pattern of pregnant sows and there is no data to the best of our knowledge to compare our results with.

## Conclusion

In conclusion, feeding pregnant sows earlier in the morning (0730 hours) appear to minimize sows’ FAA, daily feeding, and total activity, but similar cortisol response relative to pregnant sows fed daily at 1130 and 1530 hours. Additionally, feeding sow daily at 1130 hours resulted in greater FAA, feed, and total activities but reduced cortisol concentration. This study suggests that elevated sow activity might not necessarily indicate activation of hypothalamic–pituitary–adrenal axis or stress response. Finally, feeding pregnant sows once daily at 0730 hours may improve sow behavior relative to feeding at 1130 and 1530 hours.

## Abbreviations

AUC: area under the curve
FAA: feed anticipatory activity
HPA: hypothalamic–pituitary–adrenal
RISMS: Remote Insight’s Sow Management Solution

## Limitations of study

Authors identified one limitation associated with the present study. Animals on different treatments could not be housed in different rooms in the barn. However, to reduce this expected impact on our results, we allowed 21 d adaptation to the feeding regimes. This limitation may highlight the necessity for further research.
